# Preparation of nanoemulsion with milk protein-phenolic acids-α-linolenic acid rich lipid extracted from *Lepidium sativum*: evaluation of emulsification and oxidative stability of α-linolenic acid in nanoemulsion

**DOI:** 10.1007/s13197-025-06276-1

**Published:** 2025-05-02

**Authors:** Ahmed Ali Abd El-Maksoud, Karima Said Mohamed Hammad, Bothaina Shaban AbdElHakeem, Ekram Abd El-Salam Abd El-Salam

**Affiliations:** 1 Department of Dairy Science, Faculty of Agriculture, Cairo University, Giza, 12613 Egypt; 2 Department of Food Science, Faculty of Agriculture, Cairo University, Giza, 12613 Egypt; 3 Department of Chemistry, College of Science, King Khalid University, Guraiger, Abha 62529 Saudi Arabia

**Keywords:** Nanoemulsion, Milk protein, Phenolic acid, Emulsifier, Antioxidant

## Abstract

Polyunsaturated fatty acids rich oils, especially in the form of emulsion, are susceptible to oxidation, which limits their food applications. Hence, the current study aimed to develop a novel milk protein-phenolic acid complex as an emulsifier, characterized by high antioxidant activity. Caffeic (Caff) or Pyrogallic (Pyr) acids were individually complexed with either sodium caseinate (NaCa/Caff; NaCa/Pyr) or whey protein concentrate (WPC/Caff; WPC/Pyr). The milk protein-phenolic acid complexes were characterized by FTIR, and their antioxidant properties were evaluated in vitro by DPPH and FRAP assays. The preceding complexes in addition to Caff or Pyr were individually used to prepare nanoemulsions containing 10% *Lepidium sativum* oil, which contained 31.26% α-linolenic and 20.85% oleic acid. The particle size values of various nanoemulsions varied between 102.3 and 221 nm, while their zeta potential had negative values higher than − 30 mv except those incorporated with either Caff or WPC/Pyr. Nanoemulsions prepared using WPC/Caff and NaCa/Caff showed the lowest oxidation percentage (19.84 ± 0.86 and 31.62 ± 1.21%, respectively) and creaming index percentage (1.20 ± 0.156 and 0.00%, respectively) after storage for 16 days at 4 °C. These results indicate the potential of NaCa/Caff complex as an efficient antioxidant stabilizer and emulsifier to prepare various emulsions.

## Highlights

● Creating a milk protein-phenolic acid complex to enhance the PUFA-rich O/W nanoemulsion stability.

● FTIR analysis confirmed the binding between milk protein and phenolic acids.

● Sodium caseinate/caffeic acid (NaCa/Caff) incorporating nanoemulsion showed the lowest oxidation %.

● Nanoemulsion containing NaCa/Caff complex had lowest droplet size and did not show any separation.

## Introduction

Garden cress (*Lepidium sativum*) is an edible herbal plant that belongs to Cruciferae (Brassica) family. Despite, it is originally cultivated in Egypt and Western Asian countries, it is now globally flourished. Its utilization as a source of food dates to ancient Egyptians. Oil’s content of *Lepidium sativum* (LS) seeds varies between 18.15 and 21.54% with a predominant α-linolenic (omega-3) fatty acid (Jain et al. 2016).

Nanoemulsions are thermodynamically unstable dispersed systems that usually contain spherical nanosized droplets (100–600 nm) of an immiscible liquid or liquids. Although high-pressure homogenization and ultrasonication are the two main high-energy emulsification methods that are used to prepare nanoemulsions, ultrasonication is preferred due to its simplicity and economy (Aswathanarayan and Vittal 2019). Nanoemulsions are unique systems that have great abilities to deliver various hydrophobic food components such as antioxidants, coloring agents, flavors, and nutraceuticals (Das et al. 2020). Thus, literature is recently rich with studies that concerned with the formulation of nanoemulsion and the factors affecting their stability (Chen et al. 2020).

Omega-3 fatty acids are important nutraceuticals; however, adding them to foods and beverages encountered many difficulties such as low oxidative stability, slight water solubility, and unsteady bioavailability. In this regard, Walker et al. (2015) reported that the auspicious way to combine omega-3 fatty acids into liquid food systems such as sauces, beverages, and dressing is to emulsify them in the form of nanoemulsions. On the other hand, the same authors mentioned that lipid oxidation, physical stability, and product palatability were main hindrances to adding omega-3 fatty acids in foods. Therefore, several studies have been conducted to overcome these drawbacks (Abd El-Maksoud et al. 2018, 2019).

Throughout these studies, various substances have been examined to enhance the physical stability of nanoemulsions. Among these substances, protein molecules exhibited good stabilizing tendency of oil droplets and air bubbles, which could be attributed to their unique amino acid sequences (O’Sullivan et al. 2017). Xue and Zhong (2014) found that sodium caseinate exhibited excellent emulsifying and stabilizing properties especially in the presence of soy-lecithin. They ascribed these properties to the rapid adsorption of these surfactants on the surface of droplets during processing. In this context, Smułek et al. 2021 studied the effect of whey protein isolate as an emulsifier (stabilizer) in hempseed oil nanoemulsion.

The aforementioned studies showed the good emulsifying activities of milk proteins. On the other hand, rapid lipid oxidation tendency of emulsified foods, that ascribed to high surface area of these systems, make antioxidant activity at interface to be crucial for developing new emulsified products (Kiokias et al. 2020). We hypothesized that incorporating milk protein-phenolic acid complex during nanoemulsion preparation will reduce the rancidity of LS oil and enhance antioxidant activity. As, this complex acts as both an emulsifier due to its hydrophobic interactions, and as a carrier for phenolic acids. Therefore, the current work aimed to develop a new emulsifier based on the dual functionality of this complex. The effects of complexing Caffeic or Pyrogallic acid with either sodium caseinate or whey protein concentrate on the particle sizes, oxidation stability and phase separation of the generated nanoemulsions were investigated. The synthesis of these complexes was reported; they were characterized using FTIR analysis, and their antioxidant activities were evaluated using DPPH and FRAP assays. Moreover, the synthesized complexes effect on oxidative and emulsion stabilities of omega-3 rich LS oil nanoemulsion was determined.

## Materials and methods

### Material

Garden cress (*Lepidium sativum*) was purchased from local market Cairo, Egypt. Sodium caseinate (NaCa) and Whey protein concentrate (WPC) powders were provided by Arla food industries (Aarhus, Denmark). The total protein content of NaCa and WPC powders were 89.50% and 81.58% (w/w) respectively. Pyrogallic acid (Pyr), Caffeic acid (Caff), potassium ferricyanide, ferric chloride, aluminum chloride, Tween 80 (Polyoxyethylenesorbitan monooleate), 2,2-Diphenyl-1-picrylhydrazyl (DPPH), Hexane, and thiobarbituric acid were obtained from Sigma Chemical Co., Ltd (St. Louis, MO, USA).

## Methods

### Extraction of *Lepidium sativum* (LS) seed oil

LS seeds were pulverized using bench top lab mill (Cole- Parmer, USA), then they were extracted by soaking in hexane at room temperature for 12 h. The obtained extract was transferred to EYELA rotary evaporator (N-1000, Japan) to evaporate the solvent under vacuum and the temperature of 40 °C. The obtained oil was kept in brown glass bottles and stored at 4 °C.

### Oil quality parameters

Quality parameters of LS oil in terms of acid value, peroxide value and refractive index were determined according to AOCS (2009).

### Preparation and identification of fatty acids methyl esters (FAMEs)

Cold saponification process was used to prepare the FAMEs. Agilent 6,890 series gas chromatography (Agilent technologies Inc. CA, USA) supplied with a flam ionization detector (FID) and DB-23 capillary column (60 m × 0.32 mm × 0.25 μm) was used to analyze the prepared FAMEs. The carrier gas was N_2_ which flowed at 1.6 mL/min and split ratio of 50:1. The following temperature program for column oven temperature was set as follows: the initial temperature was 150 °C which held for 1 min; then at rising rate of 10 °C/min, the temperature was risen to 170 °C and held for 5 min; finally, the oven temperature was risen to 220 °C at rising rate of 5 °C/min and held for 3 min. The injector was set at a constant temperature of 250 °C, while the detector temperature was maintained at 270 °C. Air, H_2_, and N_2_ were flowed through the detector at the rate of 450, 40, and 25 mL/min, respectively. Authenticated fatty acid standards were utilized to identify and quantify the fractionated fatty acids.

### Preparation of milk protein-phenolic acids complex

Milk protein (NaCa or WPC) / phenolic acids (Pyr or Caff) complexes were prepared according to Abd El-Maksoud et al. (2019). Milk protein (0.5%) and phenolic acid (0.02%) were dissolved in Milli-Q water. The pH was risen to 10 (NaOH 0.5 M) and kept for 10 min. Then, it was lowered to 3.5 (HCl 0.5 M) and left for 10 min. Afterward, the pH was adjusted to 7. Unreacted phenolic acids were eliminated by dialyzing the mixture in a dialysis tube with an 18 K Dalton cut-off against Phosphate buffer saline (pH 7.0). The ability of the obtained milk protein/phenolic acids (electrostatic binding) complexes to stabilize and emulsify LS oil-in-water (O/W) nanoemulsions was assessed.

### Antioxidant activity

The antioxidant activity of various milk protein-phenolic acid complexes in comparison to phenolic acids were determined using DPPH and FRAP assays as outlined below:

**DPPH radical scavenging activities** of various samples were determined spectrophotometrically (Unico UV-2000, USA) at 517 nm according to Abd El-Salam et al. (2020). The DPPH inhibition percentage was calculated by the following equation:

1 $$\eqalign{&DPPH\,{\rm{ }}inhibition{\rm{ }}\,percentage \cr& = {\eqalign{& {A_{517nm}}ofDPPH\,solution \cr& - {A_{517nm}}ofDPPH\,and\,extract\,solution \cr} \over {{A_{517nm}}ofDPPHsolution}} \times 100 \cr} $$

**Ferric reducing antioxidant power (FRAP)** was determined according to the method of Hinneburg et al. (2006) with mild modification. In a test tube, 1 mL of a sample with various concentration was added to 2.5 mL of potassium ferricyanide (1%) and 2.5 mL of phosphate buffer (pH 6.6, 0.2 M). The tube was then vortexed and incubated at a constant temperature of 50 °C for 20 min. Afterwards, 2.5 mL of trichloroacetic acid (10%) was transferred to the tube and vortexed again. Later, 2.5 mL of the preceding mixture were mixed with 2.5 mL of distilled water and 0.5 mL of ferric chloride (0.1%), finally the absorbance was measured at 700 nm. Increasing absorbance of the reaction mixture indicates increasing reducing power. The FRAP of various samples were expressed in terms of IC_50_, which means the extract concentration resulted in the absorbance equal to 0.5.

### Fourier transform infrared (FTIR) spectroscopic

FTIR spectra of various samples in the range 4000–400 cm^− 1^ were determined using an FTIR spectrophotometer (Thermo scientific Nicolet 380, USA) at room temperature with a resolution of 4 cm^− 1^.

### Nanoemulsion preparation

LS oil (10-mL), 0.2-mL tween 80, and 89.8-mL milk protein-phenolic complex (mentioned above) were mixed on a benchtop magnetic stirrer (Medline Scientific, Model MS 300, UK). Then, they were sonicated (Ultrasonics, USA) for 10 min at 80% of a maximum power output of 500 W. The apparatus probe (3 mm diameter) was immersed 30 mm below the mixture surface. The sample was placed in an iced-water bath to control the heat that is generated during the emulsification process.

### Determination of droplet size and zeta potential

The mean particle size of oil droplet, polydispersity index (PDI) and zeta potential of nanoemulsions were estimated by Zeta sizer Nano ZS (Malvern Instruments, Worcestershire, UK). The estimation was done instantly after nanoemulsions preparation, where the samples were diluted with deionized water at the ratio of 1:100. The zeta potential was measured as a mean of triplicate measurements.

### Thiobarbituric acid reactive species (TBARS)

Thiobarbituric acid reactive species (TBARS) assay was implemented to assess the inhibitory ability of milk protein-phenolic complexes against lipid oxidation within the emulsion system as described by Abd El-Maksoud et al. (2018). To accelerate the oil oxidation, 250 µl of FeSO_4_ (25 mM) were added to 2 mL of the emulsions and incubated with stirring for 15 min at ambient temperature. Then, 1 mL of the previous mixture was mixed well with 2.0 mL of TBARS reagent (0.375% w/v thiobarbituric acid and 15% w/v tricholoracetic acid in 0.25 M HCl) in a test tube which then put into a water bath at 100 °C for 15 min. The tubes were chilled down and centrifuged at 4000 rpm (Hettich Rotanta 460R centrifuge, Germany) for 2 min. The absorbance was measured at 532 nm after 10 min, using UV visible spectrophotometer (Unico UV-2000, USA). The calculation of oxidation degree was calculated according to Eq. (2):

2 $$Oxidation{\rm{ }}\left( \% \right){\rm{ }} = {{{{\rm{A}}_s}} \over {{{\rm{A}}_0}}} \times 100$$

where the A_s_ and A_0_ are the absorbance of nanoemulsions containing milk protein phenolic complexes and control (nanoemulsions containing only Tween 80) samples, respectively.

### Creaming index

Creaming index of various samples was determined according to method of Yerramilli and Ghosh (2017). The samples were transferred to clear, screw glass test tube and stored under cooling (4 C°). The lower layer (serum layer height (mm) in each emulsion was visually observed and recorded after 2, 4, 8, and 16 storage days. The creaming index (CI) as a measure of the occurrence of phase separation was calculated as outlined in Eq. (3)

3 $$Creaming{\rm{ }}\:Index{\rm{ }} = {{{\rm{Serum\:layer\:height}}\left( {{\rm{mm}}} \right){\rm{}}} \over {{\rm{total\:emulsion\:height}}\left( {{\rm{mm}}} \right)}} \times 100$$

### Transmission electron microscopy (TEM)

The microstructure of LS nanoemulsion were evaluated using TEM (JEOL, JEM 1400 Flash, Japan). A 2–5 µL drop of the sample was placed on a Carbon - coated 400 mesh copper grids. Excess sample was wicked away with filter paper. Afterward, the sample was negatively stained with a 20 µL drop of 2% w/v PTA (Phosphotungstic acid) for 30–60 s and the grid was allowed to air dry. Grid was examined at an accelerating voltage of 80 kv (Mazarei and Rafati 2019).

### Statistical analysis

The chemical analyses were carried out in triplicate and the obtained results are presented as the average of the obtained values ± standard deviation. The data were subjected to analysis of variance (ANOVA) which followed by Tukey’s test. The value of *p* < 0.05 was considered statistically significant. Analyses were performed using XLSTAT software.

## Results and discussion

### *Lepidium sativum* seed (LS) oil characteristics and its fatty acid composition

The chemical and physical parameters of LS oil in addition to its fatty acid profile are recorded in Table 1. Acid value, refractive index and peroxide value of LS oil were 0.3 (mg KOH/g oil), 1.471, and 1.8 (mequiv. peroxide/ kg oil), respectively, which confirm the freshness of LS oil. The LS oil comprised eleven fatty acids, wherein the predominant fatty acid is α-linolenic acid omega-3 (31.26%). The total unsaturated fatty acids represented 81.52%, whereas the total saturated ones represented 17.02%. The same results show that the ratio between omega-6 to omega-3 (Ω-6/Ω-3) was 0.36 which promotes the protective effect of LS oil on human health (Mazarei and Rafati 2019).

**Table 1:** Physical and chemical characteristics and fatty acid composition of *Lepidium sativum* seed (LS) oil

**Physical and chemical characteristic**	
Acid value (mg KOH/g oil)	0.3
Peroxide value (mequiv. peroxide/ kg oil)	1.8
Refractive index	1.471
**Fatty acid composition (%)**	
Myristic acid (C14:0)	0.13
Palmitic acid (C16:0)	8.84
Stearic acid (C18:0)	3.18
Arachidic acid (C20:0)	3.79
Behenic acid (C22:0)	1.08
Palmitoleic acid (C14:1)	0.18
Oleic acid (C18:1)	20.85
Linoleic acid (C18:2)	11.25
Linolenic acid (C18:3)	31.26
Ecosaenoic acid (C20:1, n-9)	12.16
Erucic acid (C22:1, n-9)	6
Total saturated fatty acid (%)	17.02
Total unsaturated fatty acid (%)	81.52
Total Polyunsaturated fatty acid (%)	42.51
U/S	4.79
Ω-6/Ω-3	0.36

U/S: unsaturated fatty acid/saturated fatty acid

These results are consistent with that of Jain et al. (2016) who found that the major identified fatty acids in raw Garden cress were α-linolenic acid (27.30%) and oleic (22.90%). In addition, Umesha and Naidu (2012) found that the α-linolenic acid and Linoleic acid represented 34% and 11.8% of garden cress oil, respectively, and their Ω-6/Ω-3 ratio was 0.34.

### Fourier transform infrared (FTIR) spectroscopy

**Fig. 1:**
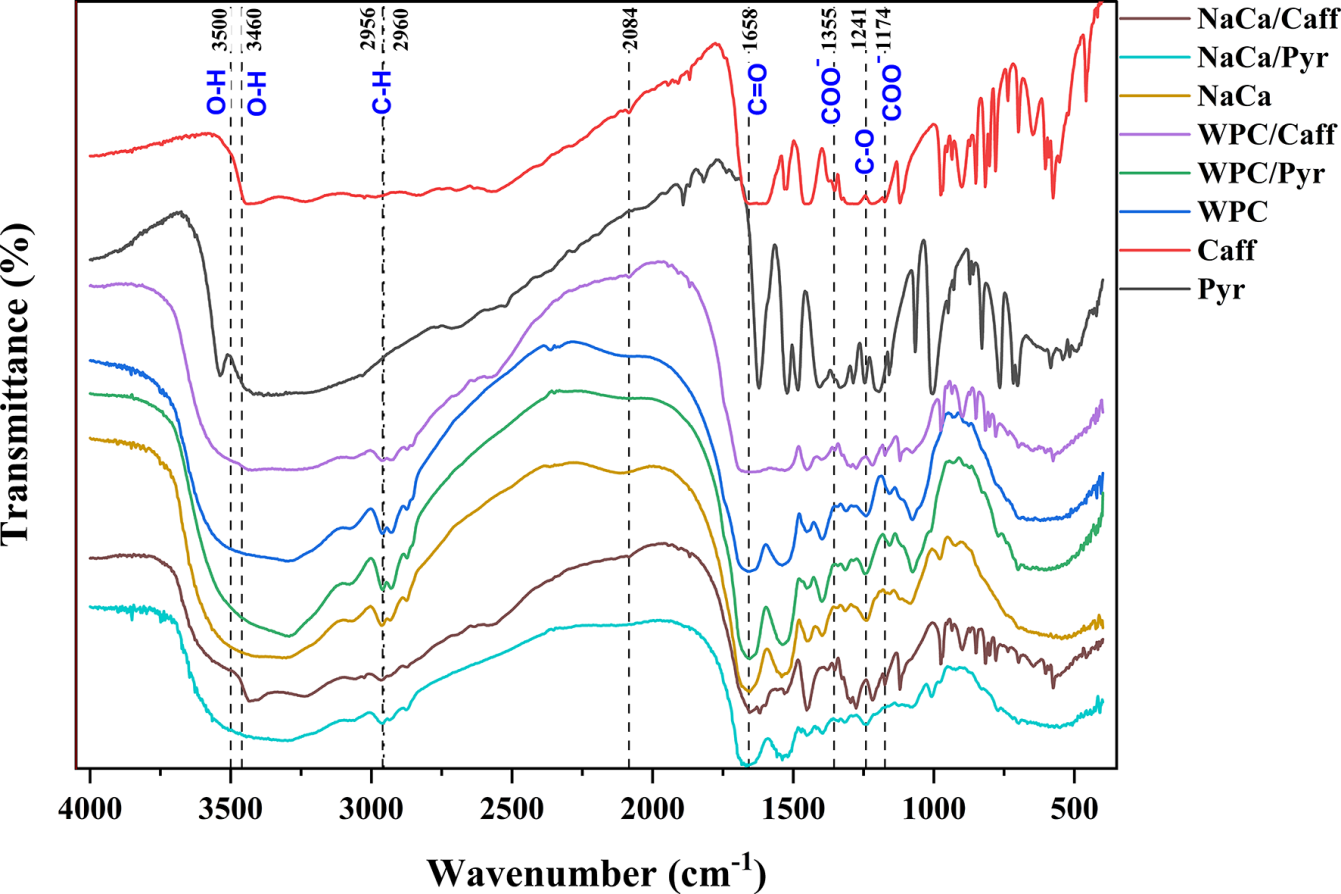
Fourier transform infrared (FTIR) spectroscopic of phenolic acids [Pyrogallic acid (Pyr), Caffeic acid (Caff)], milk protein [Sodium caseinate (NaCa), Whey Protein Concentrate (WPC) and their complex [NaCa/Pyr, NaCa/Caff, WPC/Pyr, and WPC/Caff]

FTIR was used to identify the functional groups of different samples before and after combination (Fig. 1). The spectrum of NaCa and WPC displays peaks at 2956 and 2960 cm^− 1^ respectively, representing the stretching vibrations of CH_3_, CH_2_ and CH groups in protein structure (Mihály et al. 2017). Also, the very intensity and broad peak in 3500 cm^− 1^ of milk proteins (NaCa and WPC) representing the OH groups. On the other hand, at the same wavenumber (3500 cm^− 1^) phenolic acids and their complexes exhibited sharp peaks. The spectrum of NaCas/phenolic acid complexes displays characteristic peaks at 3460, 1658, and 1241 cm^− 1^, representing the stretching vibrations of -OH groups, C = O, and C–O groups of ester, respectively (Fig. 1). Regarding the NaCa/Caff complex, the new peaks at 1174 and 1355 cm^− 1^ were assigned to the stretching vibrations of–COO groups, corresponding to the typical absorption peaks of Caff. Furthermore, the signal at 1658 cm^− 1^ attributed to the amide I due to C = O for complexes was broader than the native protein which might be an indication of successful electrostatic interaction of milk proteins and caffeic acid. Additionally, the low-intensity bands displayed at 2084 cm^− 1^ for WPC/Caff and NaCas/Caff are corresponding to the benzene ring of Caff. Yildirim-Elikoglu and Erdem (2018) reported that the most binding forces between milk protein and polyphenols is induced by reversible (weak) non-covalent forces, mostly H-bonds. The nature and strength of these interactions depends on numerous factors such as pH, temperature, polyphenol type and structure… etc. On the other hand, Muntaha et al. (2025) mentioned that polyphenols are susceptible to oxidation at basic pH (9.0), converting them to quinones which are highly reactive and can form covalent bonds with proteins through interacting with nucleophilic amino acids like lysine, cysteine, tryptophan, and methionine in protein side chains.

### Antioxidant capacity of the milk protein/ phenolic complexes

**Fig. 2:**
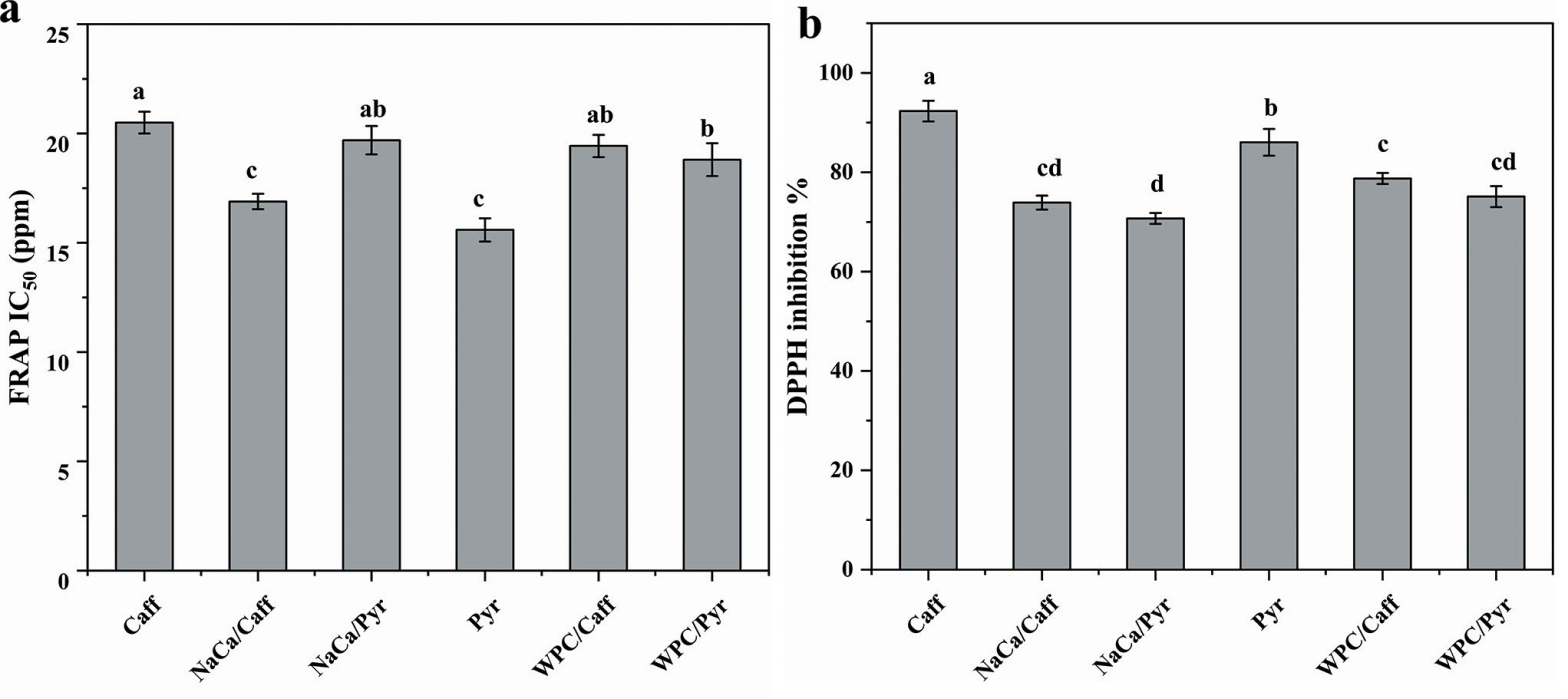
**(a)** FRAP IC_50_ and **(b)** DPPH inhibition (%)of phenolic acids [Pyrogallic acid (Pyr), Caffeic acid (Caff)], and their complexes with Sodium caseinate (NaCa) and Whey Protein Concentrate (WPC) [NaCa/Pyr, NaCa/Caff, WPC/Pyr, and WPC/Caff]

The Antioxidant capacity of various milk protein/phenolic complexes (WPC/Pyr, WPC/Caff, NaCa/Pyr and NaCa/Caff) in comparison to phenolic acids were determined in vitro using DPPH and FRAP assays as illustrated in Fig. 2. The ferric reducing antioxidant power (FRAP) assay was evolved to measure the ability of biological aqueous fluids to reduce Fe^+ 3^ to Fe^+ 2^ (Pulido et al. 2000). The simplicity of FRAP in addition to its reproducibility and capability to determine the antioxidant activity in various food matrices in comparison to more complicated methods was confirmed (Pulido et al. 2000). Thus, the FRAP of various samples was determined and the obtained results were expressed in terms of IC_50_ value as shown in Fig. 2(a). From this figure, it can be seen that solutions incorporated with Pyr only or NaCa/Caff exhibited the highest antioxidant activity, as they had the lowest significant (*p* < 0.05) IC_50_ value (IC_50_ = 15.60 and 16.89 ppm, respectively). Contrastingly, solutions incorporated with Caff only had the lowest Fe^3+^ reducing power (IC_50_ = 20.5 ppm). Pulido et al. (2000) stated that the antioxidant activity of phenolic acids relies on the number and position of their hydroxyl groups which could ascribed these variations in antioxidant activity of examined phenolic acids.

DPPH radical scavenging capacities of various samples were determined, and the obtained results illustrated in Fig. 2(b). Caff and Pyr exhibited the most significant (*p* < 0.05) DPPH radical scavenging capacities (92.95 and 88.13%), respectively. Nevertheless, milk protein/phenolic complexes showed a good antioxidant activity against DPPH radical, they significantly (*p* < 0.05) exhibited lower DPPH radical scavenging capacity than phenolic acids. Furthermore, NaCa/Caff and NaCa/Pyr complexes had the lowest scavenging capacities against DPPH radicals. These results reflect those of Abd El-Maksoud et al. (2019) who found good scavenging capacity of β-Lactoglobulin and caffeic acid complex against DPPH radicals (71.3%). In addition, Sørensen et al. (2017) found that Caffeic acid was the most efficient antioxidant in Citrem and Tween stabilized emulsions in the presence of tocopherol. On the other hand, the same authors found that Caffeic acid displayed prooxidant activity when tocopherol was not added.

### Zeta potential and particle size

Zeta potential represents the surface charge of nanoparticles, which determines the electrical repulsion forces between them. A higher zeta potential (either positive or negative) reveals strong repulsion forces leading to greater stability of nanoparticles in the suspension (Toragall and Baskaran 2021). Zeta potential values of various nanoemulsion are shown in Table 2. Except for the nanoemulsions stabilized by either Caff or WPC/Pyr complex, all nanoemulsions had high negative interfacial charges (Zeta potential > − 30 mV).

These high interfacial charges cause high repulsion force between emulsion droplets, preventing their close contact and consequently sustaining the stability of the emulsion (Mohammadzadeh et al. 2013). Actually, Yin et al. (2009) reported that the coalescence of nanoemulsions can be prevented at zeta potential of ± 30 mV, as this high electrostatic repulsion forces prevent emulsion droplets to merge. Our results are consistent with those of Chen et al. (2020). They found that nanoemulsions prepared using either whey protein isolate (WPI) or lotus seedpod procyanidins (LSPC) and their complex (WPI-LSPC) had negative interfacial charges. They ascribed these negative interfacial charges to the anionic nature of studied materials and their complexes.

On the other hand, zeta potential of any emulsion could not be able to confirm the emulsion stability without the consideration of other important factors such as phases density difference, viscosity, and the distribution of droplet size, as they with zeta potential significantly control the stability of the emulsion (Mohammadzadeh et al. 2013). In addition to its effect on stability, previous studies confirmed that droplet size of encapsulants, omega-3-rich LS oil in our study, as well as its structural character and morphology, had an enormous influence on encapsulants bioavailability (Arunkumar and Baskaran 2023; Toragall et al. 2020). The average droplet size and polydispersity index (PDI) of various samples were determined. The average droplet size of various nanoemulsions ranged from 102.3 nm to 221 nm. Moreover, nanoemulsion stabilized by NaCa/Caff recorded the highest average droplet size, whereas the nanoemulsion containing Caff only exhibited the lowest average droplet size. The correlation between the average droplet size and zeta potential was significant (*p* < 0.05) and positive (0.9295). These results differ than those previously found by Abd El-Maksoud et al. (2018), who did not find a clear correlation between zeta potential and the average droplet size. PDI of various samples varied between 0.420 and 0.850. PDI is regularly used as an indicator of the broadness of the particle size distribution. The small PDI values (≤ 0.2) indicate that the solution is monodispersed, whereas the high PDI values (> 0.2) indicate that the solution is polydispersed.

### Oxidation inhibition

**Fig. 3:**
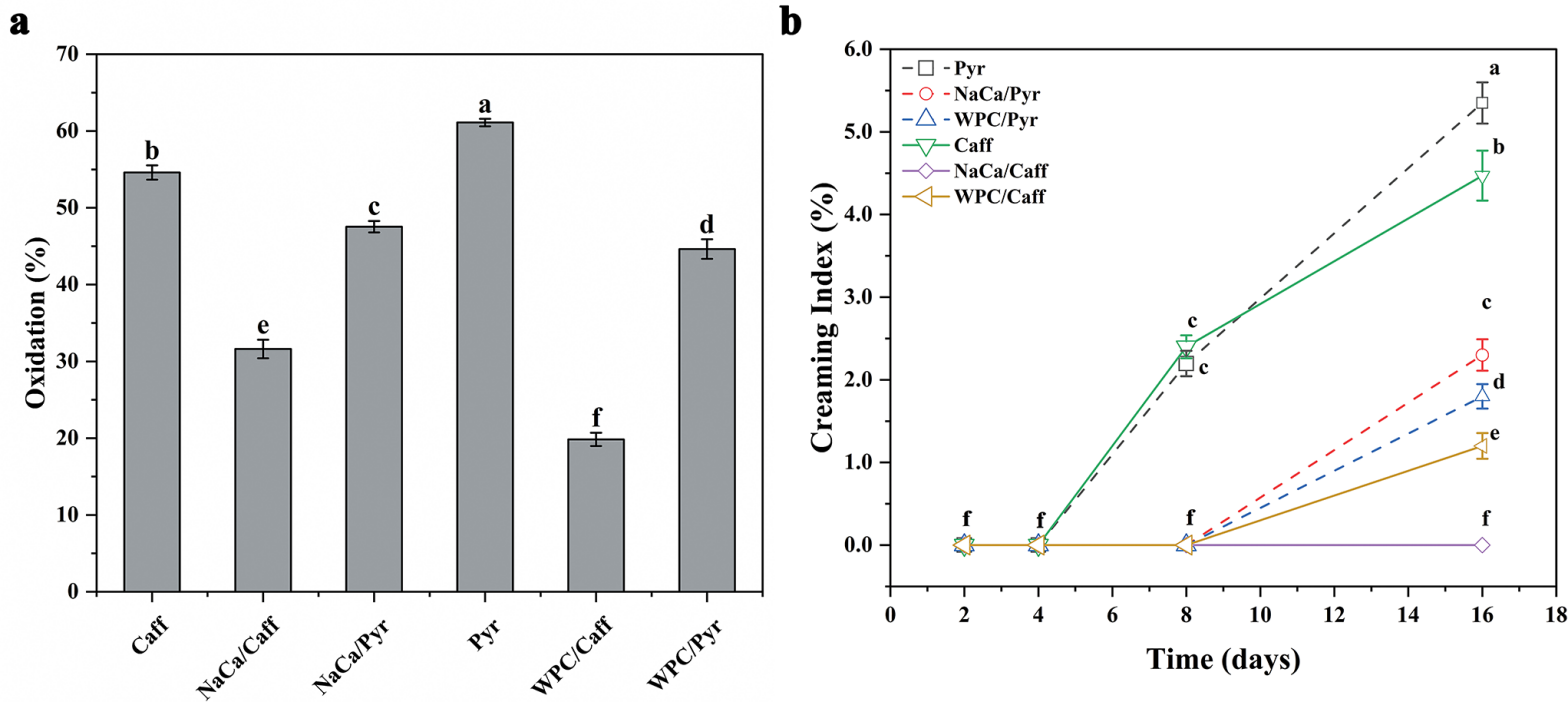
**(a)** Oxidation stability and **(b)** Creaming index of *Lepidium sativum* oil-in-water nanoemulsions prepared using phenolic acids [Pyrogallic acid (Pyr), Caffeic acid (Caff)], and their complexes with Sodium caseinate (NaCa) and Whey Protein Concentrate (WPC) [NaCa/Pyr, NaCa/Caff, WPC/Pyr, and WPC/Caff]

Oxidation susceptibility of unsaturated omega-3 fatty acids limits their uses for fortifying food products. Abd El-Maksoud et al. (2019) reported that the oxidation process is initiated at the interface of emulsified lipids where the reaction between them and free radical occurred and catalyzed in the presence of transition metals. Thus, the ability of various phenolic acids and their complexes with milk proteins to inhibit the oxidation of lipids in prepared nanoemulsions, that induced by FeSO_4_, was determined using TBARS method. Looking at Fig. 3(a), it is apparent that emulsion stabilized using either Caff or Pyr acid significantly (*p* < 0.05) showed the highest oxidation percentage. In contrast, the phenolic acids/milk protein complexes significantly (*p* < 0.05) demonstrated lower oxidation percentages. These results showed that complexing phenolic acids with milk proteins increased emulsion stability and prevented oxidation more efficiently than phenolic acids alone. The reduction in oxidation could be ascribed to antioxidant activity of either whey or casein protein (Chen et al. 2017) which in addition to phenolic acids increased the system ability to overcome the occurrence of oxidation. LS oil emulsion stabilized using WPC/Caff complex showed the lowest oxidation percentage. Indeed, WPC/Caff complex significantly reduced the oxidation by 3.08 and 2.75 folds in comparison to emulsions stabilized only by pyrogallic and Caffeic acid, respectively.

### Creaming index

Creaming index (CI%) as a measure of phase separation is usually used to determine the emulsion stability during storage time. Figure 3(b) represents the CI% of various LS nanoemulsion formulations along storage time (2, 4, 8, and 16 days at 4˚C). The CI% of nanoemulsion samples containing either Caff or Pyr acids only exhibited significant (*p* < 0.05) increase after 8 days, whereas the other emulsions showed an increase in CI% after 16 days. Moreover, at the end of storage time (16 days), the nanoemulsion containing Pyr only exhibited the highest CI% (5.35%) while the lowest CI% was observed in nanoemulsion stabilized by NaCa/Caff. This outcome is contrary to those of Yerramilli and Ghosh (2017) who found that using sodium caseinate did not able to stabilize canola oil nanoemulsion as the phase separation occurred after few hours from emulsion preparation. This variation could be ascribed to the presence of Tween 80 (emulsifier) and to low sodium caseinate concentration. As Yerramilli and Ghosh (2017) found that increasing sodium caseinate concentrations from 2.5 to 10% increased creaming index and decreased the required time for phase separation. In addition, adding Tween 80 during preparation of LS oil nanoemulsion may improve its stability through increasing steric forces between emulsion droplets (Toragall et al. 2023). Abd El-Maksoud et al. (2019) observed that utilizing β-lactoglobulin-poly Caffeic acid as emulsifier in 20% fish oil-in-water emulsion led to creaming index of 3.5% after storage for 7 days. Moreover, Abd El-Maksoud et al. (2018) found that fish oil emulsion stabilized by β-lactoglobulin caffeic acid complex at pH 6 showed creaming index of 8.13% at the end of storage time (7 days at 4 °C), whereas at the same time native β-lactoglobulin showed CI of 6.85%.

### Transmission electron microscopy

**Fig. 4:**
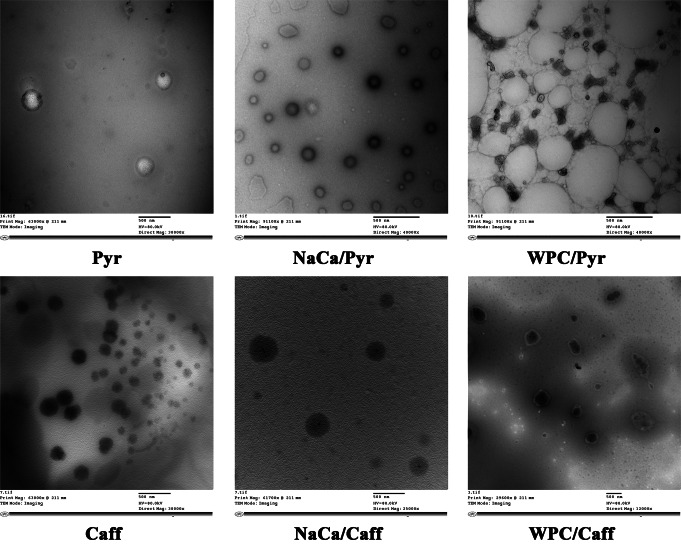
TEM of nanoemulsions prepared using phenolic acids [Pyrogallic acid (Pyr), Caffeic acid (Caff)], and their complexes with Sodium caseinate (NaCa) and Whey Protein Concentrate (WPC) [NaCa/Pyr, NaCa/Caff, WPC/Pyr, and WPC/Caff]

Droplets morphology of immediately prepared LS nanoemulsions was examined using transmission electron microscopy (TEM) as shown in Fig. 4. The LS nanoemulsions manifested heterogenic droplet sizes that had droplet diameter greater than 100 nm. These images confirm the above results of droplet size and PDI of various LS nanoemulsion formulations, as their mean droplet sizes and PDI values were above 100 nm and above 0.419, respectively, which indicate the heterogenicity of droplet sizes. The nanoemulsions stabilized by sodium caseinate showed low particle size with spherical regular shape while the nanoemulsions without proteins had high particle size and an irregular shape. These results are agreement with Mohammed et al. (2020). They found that the particle size of nanoemulsion stabilized using sodium caseinate and tween 20 were higher than 100 nm and having spherical shape with mild differences.

## Conclusion

This study has examined the impact of milk protein- phenolic acid complexes on the stability and oxidation retardation of omega-3-rich LS O/W nanoemulsion. These complexes were subjected to FTIR analysis, and their antioxidant activities were determined using DPPH and FRAP assays. FTIR analysis confirmed the electrostatic binding between milk protein and phenolic acids. FRAP and DPPH assays revealed that phenolic acids exhibited higher antioxidant activities than that of milk protein- phenolic acid complexes. To prepare various LS nanoemulsions, individual milk protein- phenolic acid complexes as well as either Caffeic or Pyrogallic acid were mixed with LS oil (10%) and Tween 80, and then the mixture wase sonicated for 10 min. Milk protein-phenolic acid complexes retarded the oxidation occurrence more significantly in all investigated nanoemulsions than phenolic acids only. Nanoemulsions stabilized with NaCa/Caff did not show any phase separation after storge for 16 days at 4 °C. Except the nanoemulsions stabilized by either Caff or WPC/Pyr complex, all other nanoemulsions had high negative zeta potential (> -30 mV). The droplet size and the polydispersity index (PDI) of all investigated nanoemulsions were ranged between 102.3 and 221 nm and between 0.420 and 0.850, respectively. In general, this study provides valuable data on the influence of phenolic acids and milk protein-based complexes on the stability of nanoemulsions. A significant strength of the study is the inclusion of multiple testing methods that were rigorously designed to ensure comprehensive results. On the other hand, further studies are required to explore the effect of pH on the nature and strength of the bond formed between phenolic acids and milk proteins under study.

**Table 2:** Droplet characteristics of the nanoemulsions stabilized by milk protein– phenolic complexes

	Zeta potential (mV)	Conductivity mS/cm	PDI	Diameter (nm)
Caff	-18.0	0.776	0.668	102.3
Pyr	-33.6	0.0557	0.85	191.2
NaCa/Caff	-50.4 and − 52.7	0.0534	0.631	221
NaCa/Pyr	-46.1	0.0362	0.669	183.6
WPC/Caff	-46.6	0.0288	0.420	209.0
WPC/Pyr	-27.8	0.141	0.543	146.9

PDI: polydispersity index, Caff: Caffeic acid, Pyr: Pyrogallic acid, NaCa/Caff: Sodium caseinate/ Caffeic acid complex, NaCa/Pyr: Sodium caseinate/ Pyrogallic acid complex, WPC/Caff: Whey Protein Concentrate/ Caffeic acid complex, and WPC/Pyr: Whey Protein Concentrate/ Pyrogallic acid complex

## Data Availability

The datasets used and/or analyzed during the current study are available from the corresponding author on reasonable request.
